# New Horizons in artificial intelligence in the healthcare of older people

**DOI:** 10.1093/ageing/afad219

**Published:** 2023-12-19

**Authors:** Taha Shiwani, Samuel Relton, Ruth Evans, Aditya Kale, Anne Heaven, Andrew Clegg, Ageing Data Research Collaborative (Geridata) AI group, Aseel Abuzour, Joseph Alderman, Atul Anand, Cini Bhanu, Jonathan Bunn, Jemima Collins, Luisa Cutillo, Marlous Hall, Victoria Keevil, Lara Mitchell, Giulia Ogliari, Rose Penfold, James van Oppen, Emma Vardy, Katherine Walesby, Chris Wilkinson, Kieran Zucker, Oliver Todd

**Affiliations:** Academic Unit for Ageing & Stroke Research, Bradford Institute for Health Research, Bradford Teaching Hospitals NHS Foundation Trust, Duckworth Lane, Bradford, West Yorkshire BD9 6RJ, UK; Leeds Institute of Health Sciences, University of Leeds, Leeds, UK; Leeds Institute of Health Sciences, University of Leeds, Leeds, UK; Academic Unit of Ophthalmology, Institute of Inflammation & Ageing, College of Medical and Dental Sciences, University of Birmingham, Birmingham, UK; Academic Unit for Ageing & Stroke Research, Bradford Institute for Health Research, Bradford Teaching Hospitals NHS Foundation Trust, Duckworth Lane, Bradford, West Yorkshire BD9 6RJ, UK; Academic Unit for Ageing & Stroke Research, Bradford Institute for Health Research, Bradford Teaching Hospitals NHS Foundation Trust, Duckworth Lane, Bradford, West Yorkshire BD9 6RJ, UK; Academic Unit for Ageing & Stroke Research, Bradford Institute for Health Research, Bradford Teaching Hospitals NHS Foundation Trust, Duckworth Lane, Bradford, West Yorkshire BD9 6RJ, UK

**Keywords:** artificial intelligence, technology, health, ageing, older people

## Abstract

Artificial intelligence (AI) in healthcare describes algorithm-based computational techniques which manage and analyse large datasets to make inferences and predictions. There are many potential applications of AI in the care of older people, from clinical decision support systems that can support identification of delirium from clinical records to wearable devices that can predict the risk of a fall. We held four meetings of older people, clinicians and AI researchers. Three priority areas were identified for AI application in the care of older people. These included: monitoring and early diagnosis of disease, stratified care and care coordination between healthcare providers. However, the meetings also highlighted concerns that AI may exacerbate health inequity for older people through bias within AI models, lack of external validation amongst older people, infringements on privacy and autonomy, insufficient transparency of AI models and lack of safeguarding for errors. Creating effective interventions for older people requires a person-centred approach to account for the needs of older people, as well as sufficient clinical and technological governance to meet standards of generalisability, transparency and effectiveness. Education of clinicians and patients is also needed to ensure appropriate use of AI technologies, with investment in technological infrastructure required to ensure equity of access.

## Key Points

- Applications of AI for older people may enable early diagnosis, stratified care and improved care coordination.
- There are concerns that AI may exacerbate health inequity and infringe on the privacy and autonomy of older people.
- Collaborative design, AI-specific governance frameworks, and investment in AI education and relevant infrastructure are needed.

## Introduction

**Figure 1:**
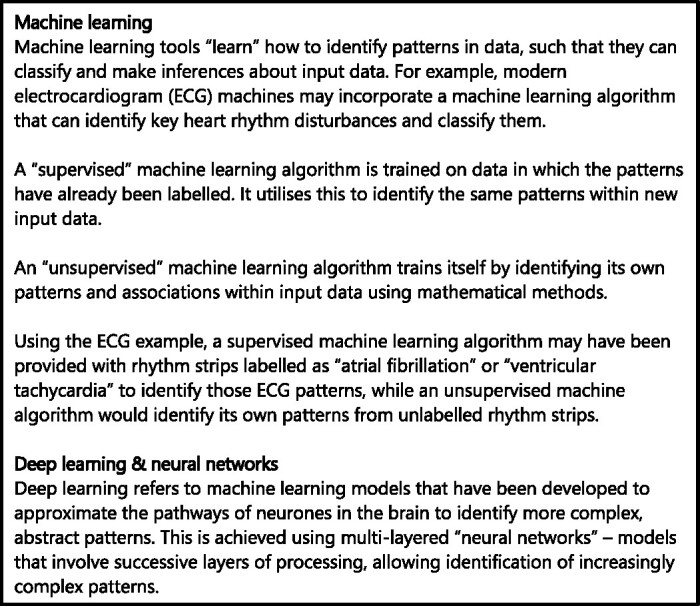
An explanation of machine learning, deep learning and neural networks, drawing on explanations provided by Sidey-Gibbons and Sidey Gibbons [2].

Artificial intelligence (AI) has been defined as any technology that simulates or surpasses human intelligence to perform a given task [1]. Most applications of AI within healthcare implement a form of ‘machine learning’, whereby computers learn from datasets to perform tasks. Machine learning models may utilise ‘deep learning’ techniques or ‘neural networks’—these concepts have been explained in Figure 1 below, adapted from a detailed explanation provided by Sidey-Gibbons and Sidey-Gibbons [2].

Global investment in AI for healthcare is increasing rapidly [3], and has been further propelled by the emergence of advanced commercial generative AI products, including chatbots like ChatGPT [4]. Within the care of older people, systematic reviews have described how AI has now been used to: assist the diagnosis and management of 21 chronic diseases of ageing [5], enable healthy ageing and maintain independence at home through monitoring and predictive technologies [6], and improve care provision and efficiency in institutional settings and in the community [7]. However, there remain significant limitations to the use of AI within the care of older people; all three reviews found the evidence for many AI technologies to be of low quality, with a lack of testing against clinical standards, and poor generalisability of many AI algorithms.

As AI governance frameworks are still developing, major governmental organisations such as the World Health Organisation (WHO) and the World Economic Forum (WEF) have warned of the risks for older people associated with misguided implementation of AI. These include infringements on their privacy and autonomy, and increased health disparities through bias in datasets and lack of access to age-specific AI technologies [8, 9]. To ensure that resources are allocated to technologies that truly benefit older people, it is crucial that all stakeholders, including older people, are involved throughout the development of AI technologies to identify key priorities and concerns [8].

### Stakeholder meetings

We held a series of four structured meetings involving a total of 41 participants including 11 Patient and Public Involvement (PPI) group members, 19 clinicians and 11 AI researchers. One week before each meeting, participants were briefed on key themes to be discussed. On the day, lunch was provided and following a presentation, themes were discussed, sometimes involving break-out groups. Within one week following each meeting, minutes and a summary of the discussion were shared to check for accuracy.

This New Horizons review is written in collaboration with the participants at these four events. The aim of this review is to increase understanding of AI technologies and to guide their development in older populations. In this review, we will explore the current landscape, challenges and future applications of AI to the care of older people.

## Where can AI provide the most benefit to the care of older people?

**Figure 2:**
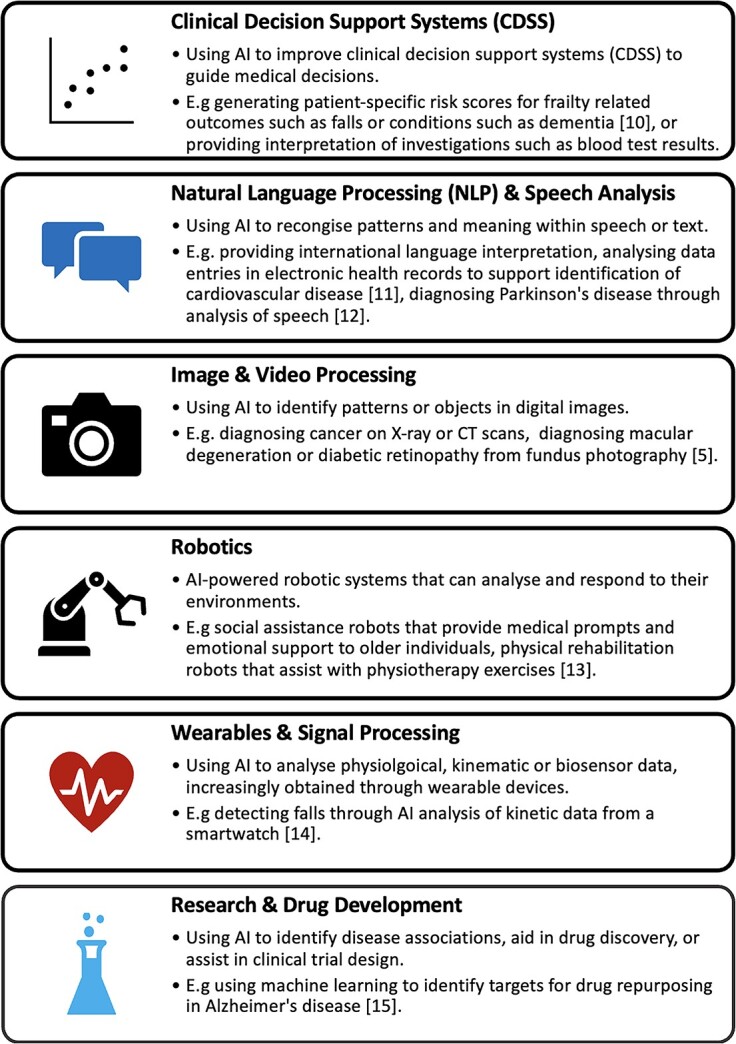
A summary of emerging applications of AI in healthcare, with examples of their use in the care of older people [5, 10–15].

There are many potential applications of AI in healthcare for older people. Figure 2 categorises some of these emerging applications.

**Figure 3:**
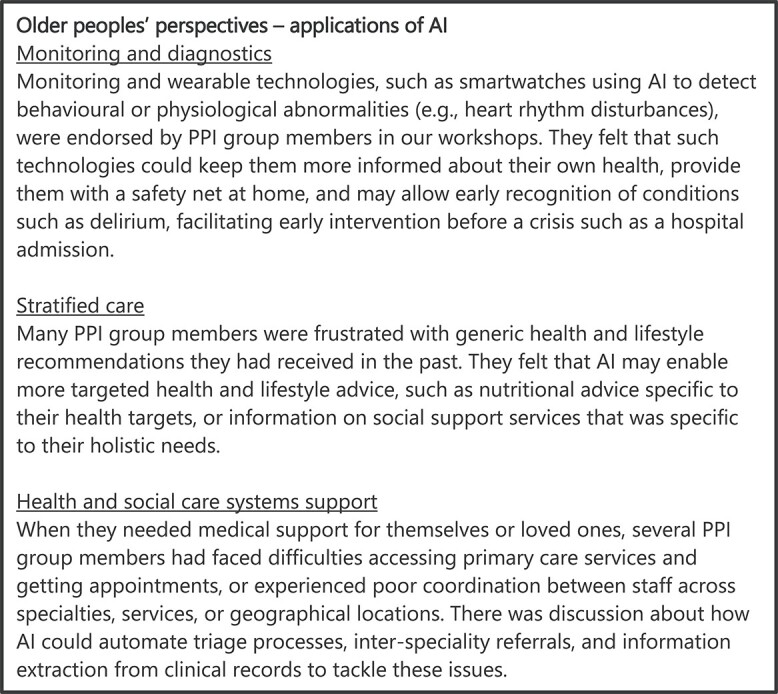
A summary of key findings from discussions with PPI group members regarding the areas of potential for AI in the care of older people.

Three areas of significant potential were identified: monitoring and diagnostics, stratified care and health systems support. Relevant discussions with PPI group members are summarised in Figure 3, and each area of care is explored in greater detail below.

### Monitoring & diagnostics

Long term conditions (LTCs) and multimorbidity disproportionally affect older people, and their accumulation with age contribute to the development of frailty and associated syndromes, such as falls, delirium, functional decline and incontinence [16]. Improved monitoring of LTCs and AI-facilitated early diagnosis may enable early intervention, reducing the burden on individuals and healthcare services.

Wearable technology or ‘wearables’ have the potential to provide continuous monitoring of multiple factors in real time in a person’s own environment. As such, they may reduce hospital admissions by supporting virtual monitoring and allowing early identification of acute deteriorations [17]. For example, a recent review of wearable-based machine learning models for predicting falls identified one with over 85% sensitivity and specificity [18]. However, it is unclear if AI-based falls prediction tools can prevent falls any more than traditional methods. Furthermore, a qualitative systematic review identified many barriers to widespread adoption of wearables among older people, including functional ability of the user (e.g. dexterity, eyesight), device features (e.g. ease of use or manipulation, aesthetics, hardware limitations), data privacy concerns, stigma and others [17].

Early recognition of frailty syndromes and LTCs may also be facilitated by AI-enabled clinical decision support systems (CDSS), image & video analysis software or natural language processing (NLP). For example, a systematic review identified several AI-assisted methods for predicting dementia [19], including:

1. Image analysis of magnetic resonance imaging scans.
2. Speech analysis of patients with Parkinson’s disease dementia.
3. A CDSS system that predicts risk of dementia using cognitive assessment scores and other clinical variables.

CDSS tools using machine learning algorithms have also been applied to the prediction of delirium [20], and to the prediction of depression among older adults [21]. Interestingly, a review in 2020 identified 35 articles studying the prediction of specific chronic diseases using AI in older people, with 9 studies claiming algorithm accuracy of at least 90% [5]. However, there was significant heterogeneity in evaluation methods, and none of the tools had been externally validated or adopted into clinical workflows [5].

### Stratified care

The term ‘stratified care’ encompasses both ‘personalised medicine’ and ‘precision medicine’ [22]. Personalised medicine typically refers to interventions tailored to an individual’s holistic needs, often using shared decision making. This accounts for factors such as an individual’s disease burden, functional capabilities, care needs and individual priorities, all of which can vary greatly amongst older people [23]. By contrast, precision medicine is often associated with ‘omics’ technologies (e.g. genomics, proteomics) and typically involves using genotypic, phenotypic or clinical data to classify individuals and target interventions based on individual, predicted responses [23]. It may be of particular value in the care of older people as they are often under-represented in population-level data that drives most clinical decisions [24]. However, the concepts overlap, and all three terms have been used interchangeably, so the term stratified care is used in this text for clarity.

By learning from an individual’s molecular, clinical and lifestyle data, AI has great potential to provide stratified care. One example is the PROTEIN (PeRsOnalized nutriTion for hEalthy living) project funded by the European Union (EU), which aims to provide personalised healthy diet plans to prevent and treat chronic disease using AI analysis of an individual’s age, lifestyle, preferences and health data [25]. Another recent study examined how AI could improve the management of hypertension, type 2 diabetes and hyperlipidaemia through stratified care [26]. Observational data from electronic health records (EHR) were used to identify cohorts of similar patients (a ‘Precision Cohort’) using a machine learning model. Treatment decisions and outcomes (blood pressure, HbA1c and cholesterol levels) from this cohort were then retrospectively evaluated to determine the optimal treatment decisions for individuals in the cohort. This was used to demonstrate that ~75% of patients could have had a better treatment option than what was provided to them through traditional practice.

However, this is a field that remains in its infancy. A systematic review published in 2022 identified a total of 28 randomised controlled trials (RCTs) of individualised interventions in older people (with or without use of AI), and very few employing data-driven analytics [27].

### Health and social care systems support

Older people are more likely to require care from multiple health and social care services, and yet they frequently have reduced access to these services [28] and receive more fragmented care, with a lack of care coordination [29]. A Canadian study identified key difficulties within care coordination for older people which apply to many healthcare systems, including fragmented information sharing between providers, difficulty identifying and referring to appropriate community services and difficulties engaging older adults in conversations about their care [30].

Several AI solutions already address some of these issues. MayaMD is a ‘health assistant’ which uses NLP to interact with users by text or voice and uses a combination of supervised and unsupervised machine learning to provide triage and telemedicine services, with a published triage accuracy comparable with that of senior clinicians, and a reported increase in user engagement [31]. AIdoc [32] and Viz.Ai [33] have both produced and validated CDSS tools designed to expedite and improve inter-specialty communication and referral processes through AI-driven recognition and referral of acute conditions, incorporating image analysis of computed tomography scans. The Democratising Access to Community Services project in Scotland is using AI to improve the ALISS tool (A Local Information System for Scotland) to provide individuals in Scotland with a wider range of personalised information on community and social services in their area [34].

A particularly promising field of research is clinical information extraction. This refers to the use of NLP to extract key information from free-text data in unstructured EHR [35]. Free-text data are estimated to make up to 80% of health data [36] and can contain valuable information, including patient diagnoses, not captured in structured data [37]. CogStack is an example of an application framework deployed in University College London Hospitals (UCLH) in the UK which uses NLP with unstructured EHR to: structure a clinician’s text in real time, extract information on diagnoses and medications, expedite clinical coding and clinical trial recruitment processes, identify patients with particular clinical phenotypes for referral pathways, and identify clinical actions (e.g. imaging orders) that have not been executed [38].

The emergence of sophisticated large language models (LLMs) such as OpenAI GPT-3.5 [4] is especially exciting. Compared with previous NLP models, LLMs are trained on vast amounts of general, textual data, and can ‘understand’ complex patterns in text and appreciate literary context. They do not require task-specific training and can also generate human-like text, enabling user-friendly technology interfaces, quick summarisations of complex clinical records and provision of clinical risk predictions among other uses [39]. However, applicability in clinical settings is currently limited by biases in training data and tendencies to fabricate information (‘hallucinations’) [40].

## What are the challenges to implementing AI technology in healthcare for older people?

**Figure 4:**
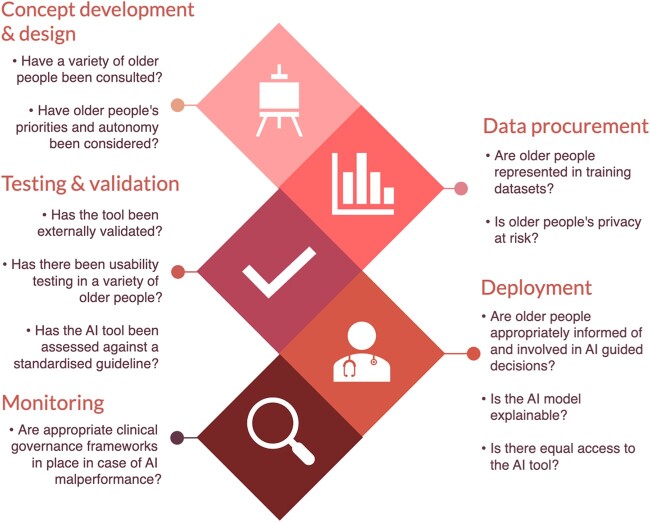
The life cycle of an AI tool and the questions that should be considered at each stage to ensure that older peoples’ values are accounted for.

**Figure 5:**
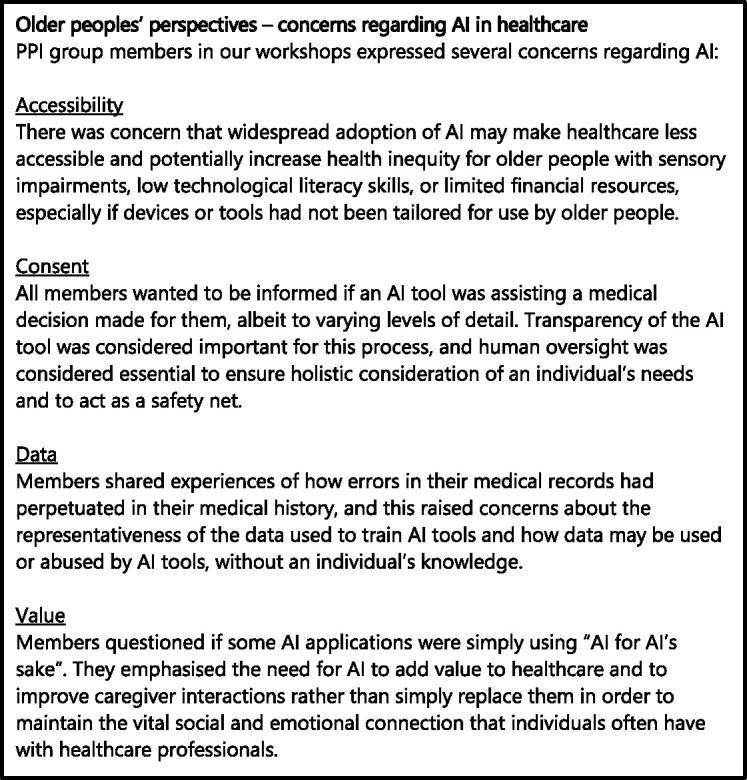
A summary of the key findings from discussions with our PPI group regarding their concerns about the use of AI in their healthcare.

If developed inappropriately, AI applications may contribute to the ‘digital divide’ of technological access, availability and efficacy between age groups. Moreover, they may compromise the rights of older people, including autonomy and privacy. These issues may be introduced at any stage of the development of an AI tool. This has been explored in detail below and summarised in Figure 4, which has been adapted from a model of the life cycle of an AI tool produced by the UK’s NHS [41]. Figure 5 summarises the discussions with our PPI group through which these concerns were explored.

### Concept development & design

Poor design of an AI tool for older people may lead to the ‘dehumanisation’ of care. This is particularly relevant for monitoring technologies, because if they replace routine caregiver interactions, they may exacerbate social isolation and reduce the possibility of opportunistic, holistic reviews [42, 43]. They may also impose behavioural standards by flagging ‘incorrect’ behaviours, eroding the boundaries between the home and medical institutions and reducing a user’s sense of autonomy and privacy. Some health monitors may additionally lead to health anxiety or stigmatise an individual by visibly highlighting their impairments [44].

More commonly, AI tools may not account for the requirements of older people. A systematic review of AI tools for older people receiving long term care found that acceptability of AI tools was mixed overall and poor for environment and wearable sensors. Wearable devices were considered uncomfortable and environment sensors could disturb sleep [7]. Acceptability would likely be even worse for technologies designed for a broader target market, potentially entrenching the digital divide across age groups [8].

To identify areas where value can truly be added to the care of older people, there is a need to adopt a user-centred design approach for AI in geriatric medicine [9]. The involvement of older people throughout the design process of individual AI technologies is crucial to this, but broader research into the perspectives of older people regarding AI technologies is also required.

### Data procurement

All AI models require a large amount of training and test data to have adequate performance. Biases within these datasets, such as under-representation of minority populations, reduce the effectiveness of AI technologies within these groups, exacerbating existing inequalities [45]. Older adults are particularly under-represented in datasets—a recent analysis of 92 publicly available datasets for AI models found that only 24 included information about the age of individuals in the dataset, with minimal inclusion of the ‘oldest-old’ (those older than 85 years) [46]. This problem has been highlighted by the United Nations (UN), which emphasised the need for transparency of data and disaggregation of data by age for data-driven decision making, especially given that most datasets represent older people as a single age cohort (e.g. older than 65 years) [47]. Further regulation in this area is required to ensure compliance with this—initiatives such as the STANDING Together initiative are already being developed to provide recommendations to manufacturers of AI tools to ensure dataset diversity, inclusivity and generalisability across demographics [48].

Maintaining the privacy of older people is also of particular concern when collecting data for AI tools. Many AI technologies designed for older people are focused at enabling ‘ageing in place’ (e.g. wearables) and can therefore collect large amounts of sensitive, personal data from patients in their own homes. A qualitative study of older people’s perception of AI technologies found data privacy to be a key concern, with older people feeling more vulnerable to privacy threats [49]. Older people with cognitive disorders or reduced technological literacy are particularly at risk [50].

Various legal frameworks exist to protect the privacy of individuals, such as the General Data Protection Regulation in the EU [51], the details of which are not within the scope of this article. However, this has not stopped poor protection of healthcare data for AI in the past, as exemplified by a UK hospital trust’s breach of data protection regulations when it collaborated with Google Deepmind [52]. Regulatory and legal frameworks for AI are developing, but in the interim, it is critical that older people are informed about issues of data protection and can control what information is shared with caregivers and third parties. Their capacity for such decision making should also be empowered. Individuals can therefore weigh privacy risks against the benefits to physical health and wellbeing afforded by AI technologies, with each person being likely to have different privacy limits [53].

### Testing & validation

As with any clinical intervention, it is important that AI tools undergo external validation and assessment of clinical utility in their target population. AI tools should clearly demonstrate added value compared with the existing standard of care. However, a recent systematic review of 65 RCTs evaluating AI prediction tools showed that nearly 40% of tools had no clinical benefit compared with standard care. In the 17 RCTs that had a low risk of bias, machine learning tools showed no benefit over traditional statistical approaches [54]. In geriatric medicine specifically, a systematic review of machine learning technologies developed for LTCs in older adults identified significant heterogeneity in how such technologies are evaluated, a lack of clinically meaningful outcome measures and a lack of testing against clinical gold standards or real-time data [5]. It is important to note that even if an AI algorithm is approved as a medical device by regulatory agencies such as the Food and Drug Administration (FDA) in the USA, the Medicines and Healthcare products Regulatory Agency in the UK or the European Medicines Agency in the EU, it may not be beneficial to patient care [55].

To ensure that AI tools are robust, there is a need for standardisation of the reporting and clinical assessment of such technologies. Goldsack et al. [56] have developed a three-stage framework for the evaluation of monitoring technologies, which includes ‘verification’ of sensor outputs, ‘analytical validation’ of physiological metrics produced from sensor outputs and ‘clinical validation’ of the technology. For AI based prediction models, a 20 question framework was published in 2020 to assess the transparency, replicability, ethics and effectiveness of AI based prediction tools [57]. A reporting guideline and risk of bias assessment tool specific to AI diagnostic and predictive models is also now in development (the TRIPOD-AI and PROBAST-AI tools, respectively) [58]. Importantly, AI tools for older people should be assessed according to outcomes identified as important by older people themselves, including factors such as autonomy and control, loneliness and isolation, and activities of daily living [59].

### Deployment

Even if robust and validated AI tools are developed, many AI-enabled decision aids, particularly those using deep neural networks with multiple complex layers of hidden processing, may not be ‘explainable’; it may not be clear why a model recommends a particular clinical decision from given input data [60]. Patients and clinicians may not trust such decisions, and it has been argued that they undermine the ability to provide informed consent, as AI tools will always have some level of error and bias, and decisions need to be contextualised according to the unique circumstances, priorities and needs of individual patients [61]. This is particularly important in the care of older people and those with multiple chronic diseases, as disease-specific outcomes may be less important than outcomes such as independence, emotional wellbeing and symptom burden [62]. Developing trust in AI therefore requires more explainable AI tools, as advocated by the EU [1], or at the least, clear disclosure of the use of a ‘black-box’ AI system. A recent systematic review published in 2022 highlighted both the progress that has been made in developing explainable AI methods for healthcare and areas that require more attention, such as explainable AI methods for identifying key text in clinical notes [63]. However, human oversight may always be needed to ensure that decisions are patient-centred and align with individual patient’s values [64].

The EU also advocates strongly for equity of access to AI-enabled systems [1]. Although use of technology among older people is increasing, older people are still less likely to have access to a computer or the Internet [65], particularly those from lower socioeconomic groups and minority ethnic groups in the UK and the USA [66, 67]. There is a risk that AI systems may widen disparities in health outcomes, both between groups of older people, and between older people and the wider population, based on relative access to AI-enabled technology. This is recognised by the WHO which recommended that an investment in digital infrastructure and technological literacy for older people is required to prevent ageism in AI for health [8].

### Monitoring

It is vitally important that AI tools undergo close monitoring when they are deployed in different real-world settings, as their performance is highly dependent on the data provided to them in different contexts, and they will inevitably make errors. There is also a well-established risk of performance decay with time in a dynamic healthcare setting, as many algorithms are not allowed to be modified after approval (as required by organisations such as the FDA) [68]. Research on automation technology suggests that older adults, particularly those with cognitive decline, may be particularly susceptible to over-reliance on AI tools and less likely to detect such errors [69]. Safeguarding older adults, particularly those who are more vulnerable, will require robust clinical governance frameworks, regular impact assessments and audits, and specific attention from governmental and regulatory bodies [8].

## The future of AI in the care of older people

AI has the potential to improve the patient experience within the healthcare system, allowing older people to live at home for longer (‘ageing in place’), enabling stratified care, and increasing timely access to health and social care services. However, while many AI applications are in development for older people, few have been externally validated, or robustly evaluated in clinical practice.

Future research should ensure that AI tools are co-developed by AI experts, health and social care providers, policy makers and older people to identify how AI could add value without sacrificing the autonomy, privacy, and social or emotional health of older people. This is also important to ensure appropriate resource allocation; resources should be spent on developing AI tools that are likely to be effective and impactful, rather than developing large numbers of AI technologies that have lower utility. Robust clinical and technological frameworks are needed as well to ensure the generalisability, transparency and effectiveness of AI tools that are developed, including guidelines on data procurement, privacy protection, AI model transparency and post-deployment monitoring. Geriatricians and members of the wider multidisciplinary team providing care for older people will need training in AI methodology to appraise the use of such frameworks, to obtain informed consent from their patients and to advocate for the inclusion of older people in AI datasets and evaluation of AI tools. To ensure engagement with and support for AI-based approaches, there is a need to invest in AI literacy and access to technology for older people as the key potential beneficiaries from novel AI-based approaches to care.
